# Genome-wide identification and analysis of the cytokinin oxidase/dehydrogenase (*ckx*) gene family in finger millet (*Eleusine coracana*)

**DOI:** 10.3389/fgene.2022.963789

**Published:** 2022-09-27

**Authors:** Rostyslav Blume, Alla Yemets, Vitaliy Korkhovyi, Volodymyr Radchuk, Dzhamal Rakhmetov, Yaroslav Blume

**Affiliations:** ^1^ Department of Population Genetics, Institute of Food Biotechnology and Genomics, National Academy of Sciences of Ukraine, Kyiv, Ukraine; ^2^ Department of Cell Biology and Biotechnology, Institute of Food Biotechnology and Genomics, National Academy of Sciences of Ukraine, Kyiv, Ukraine; ^3^ Leibniz Institute of Plant Genetics and Crop Plant Research, Gatersleben, Germany; ^4^ M. M. Gryshko National Botanic Garden of National Academy of Sciences of Ukraine, Kyiv, Ukraine; ^5^ Department of Genomics and Molecular Biotechnology, Institute of Food Biotechnology and Genomics, National Academy of Sciences of Ukraine, Kyiv, Ukraine

**Keywords:** cytokinin dehydrogenase/oxidase enzymes, *EcCKX* genes, phylogeny, gene expression, *Eleusine coracana*, finger millet, food security

## Abstract

Cytokinin dehydrogenase/oxidase (CKX) enzymes play a key role in regulating cytokinin (CK) levels in plants by degrading the excess of this phytohormone. *CKX* genes have proven an attractive target for genetic engineering, as their silencing boosts cytokinin accumulation in various tissues, thereby contributing to a rapid increase in biomass and overall plant productivity. We previously reported a similar effect in finger millet (*Eleusine coracana*) somaclonal lines, caused by downregulation of *EcCKX1* and *EcCKX2*. However, the *CKX* gene family has numerous representatives, especially in allopolyploid crop species, such as *E. coracana*. To date, the entire *CKX* gene family of *E. coracana* and its related species has not been characterized. We offer here, for the first time, a comprehensive genome-wide identification and analysis of a panel of *CKX* genes in finger millet. The functional genes identified in the *E. coracana* genome are compared with the previously-identified genes, *EcCKX1* and *EcCKX2*. Exon-intron structural analysis and motif analysis of FAD- and CK-binding domains are performed. The phylogeny of the *EcCKX* genes suggests that *CKX* genes are divided into several distinct groups, corresponding to certain isotypes. Finally, the phenotypic effect of *EcCKX1* and *EcCKX2* in partially silencing the SE7 somaclonal line is investigated, showing that lines deficient in *CKX*-expression demonstrate increased grain yield and greater bushiness, enhanced biomass accumulation, and a shorter vegetation cycle.

## Introduction

Plant growth regulation depends on the balance of phytohormones, among which cytokinins play an important role. Cytokinins are plant hormones that not only take part in cell proliferation and differentiation, but also in the establishment of plant architecture. They regulate many developmental processes, strongly influence grain yield (Zhang et al., 2012; Jameson and Song, 2016; Chen et al., 2020b; Dash and Rai, 2022), and impact the plant signaling pathways of biotic and abiotic stresses (Werner et al., 2006; Blume et al., 2017). Cytokinin homeostasis is regulated by members of several multigene families through a balance between biosynthesis [isopentenyl transferase (IPT)], activation [Lonely Guy (LOG)], inactivation (O-glucosyl transferase), re-activation (β-glucosidase), and degradation [cytokinin oxidase/dehydrogenase (CKX)] (Werner et al., 2006; Jameson and Song, 2016). In particular, *CKX* genes encode the cytokinin oxidase/dehydrogenase enzymes, which catalyze the irreversible degradation of cytokinin. Such enzymes are responsible for the balance of cytokinins in the plants, and for the control of cytokinin-dependent processes (Werner et al., 2006; Dash and Rai, 2022).

It should be noted that CKXs are encoded by a relatively small gene family in different plant species, such as rice (Ashikari et al., 2005; Mameaux et al., 2012), *Arabidopsis* (Galuszka et al., 2007), maize (Gu et al., 2010; Mameaux et al., 2012; Zalabák et al., 2014), foxtail millet (Mameaux et al., 2012; Wang et al., 2014), barley, wheat, sorghum, and brachypodium (Mameaux et al., 2012), Chinese cabbage (Liu et al., 2013), potato (Suttle et al., 2014), oilseed rape (Liu et al., 2018), forage legume barrel medic (*Medicago truncatula*) (Wang et al., 2021), soybean (Liu et al., 2021; Nguyen et al., 2021), cowpea (Liu et al., 2021) and apple (Liu et al., 2022). Following phylogenetic, molecular, and comparative analyses of *CKX* families in sequenced grass species, such as rice, *Brachypodium distachyon*, sorghum, maize and foxtail millet, as well as members identified from wheat and barley transcriptomes⁄genomes, the phylogenetic analyses identified four Poaceae *CKX* clades (Mameaux et al., 2012). Comparative analysis showed that such phylogenetic groupings of *CKX* can largely be explained by a combination of local gene duplication and a whole-genome duplication event that preceded their speciation. Recently, the evolutionary origin of CKXs has been analyzed and attempts have been made to understand their function in relation to their structure in different organisms (Dabravolski and Isayenkov, 2021).

Even monocots and dicots show significant differences in cytokinin metabolite composition (Jiskrová et al., 2016). Inhibition of *CKXs* can lead to increased plant productivity, and thereby increased crop yields (Zhang et al., 2012; Jameson and Song, 2016; Szala et al., 2020). These changed features were detected by us earlier in finger millet (*Eleusine coracana*) (Radchuk et al., 2012). We found that downregulation of two *CKX* genes (i.e., *EcCKX1*, *EcCKX2*) in the obtained somaclonal variants of *E. coracana* leads to the formation of a dwarf and highly productive phenotype of finger millet (Radchuk et al., 2012). As a consequence of this gene downregulation, attenuated degradation of cytokinins in such *E. coracana* lines leads to higher cytokinin levels, increased accumulation, and stimulation of meristem activity, resulting in the production of more inflorescences and seed setting (Radchuk et al., 2012; Yemets et al., 2020). Similar data for rice were previously reported by Ashikari et al. (2005), where the authors found that a decrease in *OsCKX2* expression leads to the accumulation of cytokinin in the meristems of inflorescences, an increase in the number of reproductive organs, and, as a result, an increase in grain yield. Other studies confirm such an effect: the silencing *ckx2* in barley, for example (Zalewski et al., 2014), with overexpression of this gene leading to a non-flowering phenotype (Mrízová et al., 2013). This gene is described as having the same function in *A. thaliana* (Li et al., 2013) and also in the development of seed pods in *B. napus* (Liu et al., 2018).

Given the role of cytokinins in plant development and productivity, the identification and regulation of *CKX* gene expression in main crops is extremely important from the perspective of increasing yield and food security. Due to the ability of *E. coracana* to grow in arid and semi-arid regions of Central Africa and India, as well as in tropical regions (more than 25 countries in Africa and Asia), its ability to withstand abiotic and biotic (resistance to pathogens) stresses (Gupta et al., 2017); its nutritional value (the grains are rich in amino acids, such as methionine and lysine; minerals such as calcium, iron, zinc, phosphorus, and potassium; vitamins; and fibers), and its good grain storage properties, finger millet is one of the most important crops for sustainable agriculture in developing countries, and a very valuable food resource. Finger millet is gluten free and therefore can be included in the diet of patients suffering from celiac disease (Pagano, 2006). In addition, finger millet can still potentially be used as animal fodder (Gupta et al., 2017), and for bioethanol production (Yemets et al., 2020). To date, there is no accurate information on the global cultivation of this crop, although India is known to be the largest producer, with finger millet grown on 1.19 million hectares and yielding over 1,660 kg/ha (Sood et al., 2019).

The availability of finger millet genomic resources (Hittalmani et al., 2017; Hatakeyama et al., 2018; Zhang et al., 2019b; Bančič et al., 2021; Pendergast et al., 2022) makes it possible to analyze different groups of genes and estimate practical methods for molecular breeding and biotechnological improvement of this crop (Antony Ceasar et al., 2018; Pandian and Ramesh, 2019; Sood et al., 2019). Certainly, the identification and characterization of all CKX family members in *E. coracana* is a very important tool for achieving these practical goals. In this paper, we present for the first time the comprehensive genome-wide identification and analysis of a panel of *CKX* genes in finger millet. These functional genes, identified in the *E. coracana* genome, are compared with the two previously-identified genes, *EcCKX1* and *EcCKX2*. Exon-intron structural analysis, and a motif analysis of the FAD- and CK-binding domains are carried out. Study of the phylogeny of *EcCKX* genes establishes that *CKX* genes are divided into several distinct groups, according to their specific isotypes. Finally, the phenotypic effect of partial silencing of *EcCKX1* and *EcCKX2* in the somaclonal line of SE7 is investigated, suggesting that lines deficient in *CKX*-expression manifest increased grain yield, higher bushiness, enhanced biomass accumulation, and a shorter vegetation cycle. The bioinformatic data obtained will prove useful for further manipulation of finger millet *CKX* genes so as to improve crop quality and yield.

## Materials and methods

### Initial identification and analysis of cytokinin dehydrogenase/oxidase genes in the *E. coracana* genome

The initial search for *CKX* genes in the *E. coracana* genome was conducted using a series of BLAST searches of the *Eleusine coracana* annotation release v1.1 (genome ID: 560), which is deposited in the Phytozome v13 database, and which contains the most recent and complete *E. coracana* genome assembly and annotation. Personal permission to use the genome data for the current study was obtained from Prof. Katrien M. Devos, as the current release of the *E. coracana* annotation has a “Reserved Analysis” status, limiting its use for publication.

The BLAST algorithm was used to search the translated nucleotide databases, and the coding regions of the *OsCKX* and *AtCKX* genes (Swarbreck et al., 2008) were used as queries. In addition, sequences of two previously identified genes, *EcCKX1* (HE800184.1) and *EcCKX2* (HE800185.1) of *E. coracana*, were used for the genomic search (Radchuk et al., 2012). Search parameters were: E-value threshold—1e^−5^, comparison matrix—BLOSUM62, and word length—3 (Henikoff and Henikoff, 1992; Altschul, 1993). We analyzed the results and discarded short and insignificant hits.

Information on *E. coracana CKX* genes, including location, genomic coordinates, sequence ID, genomic sequence, protein sequence, and coding sequence (CDS), was acquired from the Phytozome v13 database. Orthologous information for the identified genes was retrieved from the KEGG database (https://www.genome.jp).

### The genomic organization and synteny of cytokinin dehydrogenase/oxidase genes

A multiple alignment CDS sequence of the *CKX* genes was performed using the MUSCLE algorithm (Edgar, 2004). Representations of the exon-intron structure of the genes were obtained using the Gene Structure Display Server (http://gsds.gao-lab.org/) (Hu, 2015).

The domain organization of CKX peptides was analyzed using the Pfam tool (https://pfam.xfam.org/) (Mistry et al., 2021), which allowed us to confirm the presence or absence of key FAD- and CK- binding domains. Calculation of the rate of non-synonymous substitution (K_A_), the rate of synonymous substitutions (K_S_), and their ratio (K_A_/K_S_), were performed in the TBtools v1.0971 software (Chen et al., 2020a), using CDS sequences of the identified *EcCKX*.

The organization of CK-responsive *cis*-elements was analyzed in the 2 kbp upstream regions of the identified *EcCKX* genes (taking into account the initiation codon), using the PLACE v30.0 tool (https://www.dna.affrc.go.jp/PLACE) (Higo et al., 1999), and the results were compared with the search results in a similar database—PlantCARE (http://bioinformatics.psb.ugent.be/webtools/plantcare/html/) (Lescot et al., 2002). Further data on the presence of particular *cis*-elements motifs were filtered to identify only the CK-responsive elements. The structure of the protein domain organization, and the allocation of CK-responsive *cis*-elements were visualized using TBtools v1.0971 software (Chen et al., 2020a).

Syntenic relationships between homeologous *CKX* genes from different subgenomes of *E. coracana* were analyzed in TBtools v1.0971 software (Chen et al., 2020a), using the MCScanX algorithm (Wang et al., 2012). The results were further visualized as a circos plot.

To explore the syntenic relationships of *E. coracana* orthologous *CKX* genes with *O. sativa* species, the genome data and the gene annotation files of rice (GCA_001433935.1 assembly) were also downloaded from the NCBI database. The synteny analyzing dual plot graphs were constructed using the dual synteny plotter function in TBtools, while inter-genome synteny was inferred using the MCScanX algorithm.

### Phylogenetic analysis

Phylogenetic analysis was performed using MEGAX (Kumar et al., 2018). Respective amino acid sequences of CKX proteins from different species (Supplementary Table S1), including *E. coracana*, were aligned using the MUSCLE algorithm (Edgar, 2004). The CKX protein sequences of *Arabidopsis thaliana* and *Oryza sativa* were retrieved from the KEGG genome database, while those of *Setaria italica*, *Hordeum vulgare*, *Populus trichocarpa*, and *Prunus persica* were obtained from the Phytozome v13 database.

Initial isotype determination of *EcCKX* genes was performed using cds sequences as the initial data set, which was then compared to the previously described *EcCKX1* (HE800184.1) and *EcCKX2* (HE800185.1) (Radchuk et al., 2012). This “guide” tree was constructed using the neighbor joining (NJ) method with the default software settings. The NJ tree was generated with a bootstrap support of 1,000 replicates (Supplementary Figure S1).

Before the maximum likelihood (ML) phylogenetic tree was constructed, the best substitution model analysis was performed. The Jones-Taylor-Thornton model with gamma distribution rate and invariant sites (JTT + G + I) was chosen as optimal (Jones et al., 1992). The selected model was used to infer a phylogenetic tree using the ML estimation method. The initial tree was derived using neighbor-joining analysis, following the nearest-neighbor-interchange heuristic method. The number of discrete gamma categories was four; the treatment for gaps and missing sites was “use all sites.” The statistical confidence of tree topology was assessed using a bootstrap test with 1,000 replicates.

### Plant material, growth conditions, and agro-morphological evaluation

Field evaluation of the characteristics of finger millet somaclonal mutants was carried out using approx. 100 plants per wild-type (variety Tropikanka) and SE7 (registered now in Ukraine as Yaroslav-8 variety) finger millet lines. Approximately 15–25 seeds were planted in 20-cm diam. pots in a greenhouse at the Leibniz Institute of Plant Genetics and Crop Plant Research (Gatersleben, Germany) in the late April. Plants were grown for 2 weeks in a greenhouse and then some of them were transplanted to a field in Gatersleben in May. Spacing between individual plants was 20 cm × 20 cm. Plant height and yields from a random 25 plants were measured in late September 2011. Additional field trials were conducted in Kyiv, Ukraine, at the National Botanical Garden experimental field, under the conditions described earlier (Rakhmetova et al., 2020). Plants were harvested manually and the following parameters were recorded at the plant maturity stage: plant height, 1000-grain weight, number of primary and secondary stems per plant, dry matter content (w/w), dry biomass yield, and seed yield. The obtained data were also extrapolated to rate per hectare.

### Tissue sampling for expression analyses

For quantitative reverse transcription-PCR (qRT-PCR) and northern blot analyses of different tissues, total RNA was isolated 4 and 5 days after imbibition (4 and 5 DAI), from the young leaves of seedlings at the tillering stage, and from the old leaves of maturating plants. In addition, tissue samples of the developing inflorescence were taken at three stages: Stage A at a length of 2–3 cm; Stage B at 3–4 cm; and Stage C at > 4 cm. All samples were collected at least in triplicate from biologically-independent plant material.

### RNA extraction

Total RNA was extracted from different tissues of the wild type and SE7 mutant of finger millet using Trizol reagent (Invitrogen). To do this, 100 mg of tissue was ground in liquid nitrogen, mixed with 1 ml of preheated Trizol (60°C) for 5 min, and centrifuged at 13,000 rpm for 10 min at 4°C. The supernatant was transferred into a new tube, mixed with 0.2 ml of chloroform for 2 min, and centrifuged at 13,000 rpm for 10 min at 4°C. The aqueous phase was transferred into a new tube and mixed with 0.6 vol. of isopropanol, incubated at room temperature for 10 min, and then centrifuged at 13,000 rpm for 10 min at 4°C. The pellet was rinsed once with cold 70% ethanol and dissolved in 100 µl of distilled water. The isolated RNA was treated with RNase-free DNase (Qiagen), purified using a RNeasy plant mini kit (Qiagen), and used for the synthesis of cDNA, quantitative RT-PCR, and cDNA array.

### Expression analyses

The procedure for cDNA synthesis was identical to that described previously (Radchuk et al., 2012). cDNA fragments of the *EcCKX1.A/B* and *EcCKX2.A/B* genes were further amplified from different tissues of wild-type and mutant lines of finger millet by RT-PCR, using gene-specific primers selected from conserved regions of the corresponding sequences. For distinguishing of *EcCKX1.A/B* genes, the following primers were used: EcCKX1.A/B-For—5′-TGCGCCTCGACGGCCATTTCAG-3′ and EcCKX1. A/B-Rev—5′-GGATCGTACGATTCGCCCTTCC-3′, while for *EcCKX2.A/B* genes — EcCKX2.A/B-For—5′-CACCACCATCGCTGCGTCCAGT-3′ and EcCKX2.A/B-polyT—5′-GTTGGGTNTTTTTTTTTTTTTTTTTTT-3´. The actin gene was an internal control, for which primers targeting barley actin were used: qactin_u—5′-ATGGTGGGGATGGGGCAGAAG-3′ and qvactin_r—5′-CTCCTCCGGGGCAACACGAA-3´. Northern blot analysis of the *EcCKX* genes transcript was performed according to the previously-described procedure (Conrad et al., 2007). For qRT-PCR, as described above, 5 µg of the total RNA isolated were used for reverse transcription by SuperScript III reverse transcriptase (Invitrogen), with an oligo (dT) primer. The resulting cDNAs were used as templates for qRT-PCR assays, which were performed as previously described (Radchuk et al., 2011). The PCR efficiency was assessed using LinRegPCR software (Ramakers et al., 2003). Analysis of RT-PCR data was performed according to the procedure described in our previous publication (Radchuk et al., 2012).

### Statistical processing of the data

The obtained data were statistically processed using OriginPro 2019b software. Deviations of all means were calculated as a standard deviation (SD). To reveal the significance of differences in various parameters between the studied genotypes, one-way ANOVA was used, which included the calculation of Fisher’s least significant differences (LSDs). The LSDs were used to determine homogeneous groups for values of specific morphological or productive parameters at different level of significance, *p* < 0.05, *p* < 0.01, and *p* < 0.001.

## Results

### The cytokinin dehydrogenase/oxidase gene family in finger millet

As the result of a thorough search of the finger millet genome, deposited in the Phytozome v13.0 database, 20 representatives of the *CKX* gene family were identified (Table 1). The length of the genes varied from 937 to 3,871 bp, and the length of the encoding peptides ranged from 243 to 609 aa. MUSCLE alignment of the previously-described *EcCKX1* and *EcCKX2* (Radchuk et al., 2012) against cds of the identified *EcCKX* panel revealed the existing orthologs of *EcCKX1* and *EcCKX2* in the finger millet genome, which were then named *EcCKX1.A*/*B* and *EcCKX2.A*/*B,* respectively (Supplementary Figure S1). Types of the encoded EcCKX proteins were determined on the basis of the phylogenetic analysis that will be described separately.

**TABLE 1:** List of the identified *EcCKX* genes in the genome of finger millet.

Gene name	Loci (Phytozome ID)	Gene length (bp)	Putative peptide length (aa)	Chr	Type
*EcCKX1.A*	ELECO.r07.1AG0046160	1925	523	1A	IIb
*EcCKX1.B*	ELECO.r07.1BG0096130	1913	523	1B	IIb
*EcCKX2.A*	ELECO.r07.2AG0128410	3,629	523	2A	IIIb
*EcCKX2.B*	ELECO.r07.2BG0183810	3,421	523	2B	IIIb
*EcCKX3.A*	ELECO.r07.5AG0375500	1978	523	5A	IIb
*EcCKX3p.B*	ELECO.r07.5BG0422940	937	243	5B	IIb
*EcCKX4.A*	ELECO.r07.4AG0310130	4,675	522	4A	IIIb
*EcCKX4.B*	ELECO.r07.4BG0341290	3,198	390	4B	IIIb
*EcCKX5.A*	ELECO.r07.1AG0035370	3,871	535	1A	IIa
*EcCKX5.B*	ELECO.r07.1BG0085420	3,839	533	1B	IIa
*EcCKX6.A*	ELECO.r07.8AG0633390	1705	414	8A	IIIa
*EcCKX6.B*	ELECO.r07.8BG0662450	3,044	609	8B	IIIa
*EcCKX7.A*	ELECO.r07.1AG0012730	2,118	529	1A	Ib
*EcCKX7.B*	ELECO.r07.1BG0061400	2038	531	1B	Ib
*EcCKX8.A*	ELECO.r07.1AG0013320	3,487	551	1A	Ib
*EcCKX8.B*	ELECO.r07.1BG0062080	3,732	554	1B	Ib
*EcCKX9.A*	ELECO.r07.2AG0106510	1862	543	2A	Ia
*EcCKX9p.B*	ELECO.r07.2BG0160030	1,106	332	2B	Ia
*EcCKX10.A*	ELECO.r07.6AG0543440	1782	541	6A	Ia
*EcCKX10.B*	ELECO.r07.6BG0496700	1,694	518	6B	Ia

All identified *EcCKX* were present in doublets and were allocated on homologous chromosomes from subgenomes A and B of *E. coracana*. As this species has an allotetraploid nature, all gene pairs were defined as homologous. In the vast majority of cases, the homologous genes possessed similar gene length and encoded highly identical peptides (92.55%–98.85%). Two genes, *EcCKX3p.B* and *EcCKX9p.B,* were of significantly reduced length, 937 bp and 1,106 bp, respectively, compared to other *EcCKX*, and thus encoded shortened peptides, 243 aa and 332 aa, respectively. It is also worth noting that four *EcCKX*, encoding different isotypes of this enzyme, were located on the same 1A chromosome (and their four homeologs on 1B). *EcCKX1.A* and *EcCKX5.A* were located relatively close at 7.11 Mbp, while *EcCKX7.A* and *EcCKX8.A*—were only 710 kbp apart. Also, *EcCKX1.A*/*B* and *EcCKX5.A*/*B* both belong to type II of CKXs, while both *EcCKX7.A*/*B* and *EcCKX8.A*/*B* are type I members. Co-location of pairs of these genes on the same chromosome could possibly correspond to their potentially paralogous nature.

### Exon-intron structure of *EcCKX*, and domain distribution in their peptides

**FIGURE 1:**
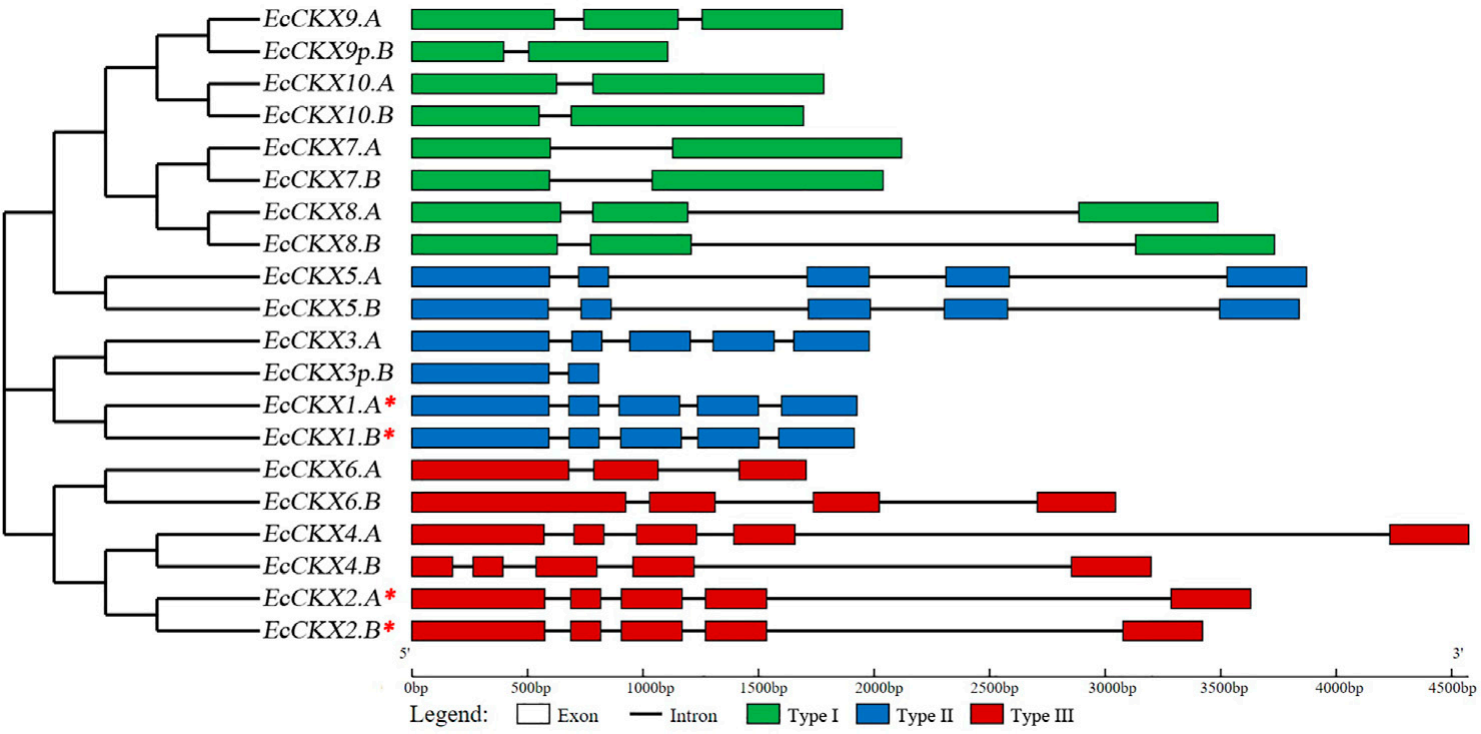
Exon-intron structure of the identified *CKX* genes within the *E. coracana* genome. The NJ phylogenetic tree was constructed with bootstrap support of 1,000 iterations (all branches had support of 65% and higher), based on cds sequences of the identified genes. A red asterix denotes genes identical to previously sequenced and characterized *EcCKX1* and *EcCKX2*.

**FIGURE 2:**
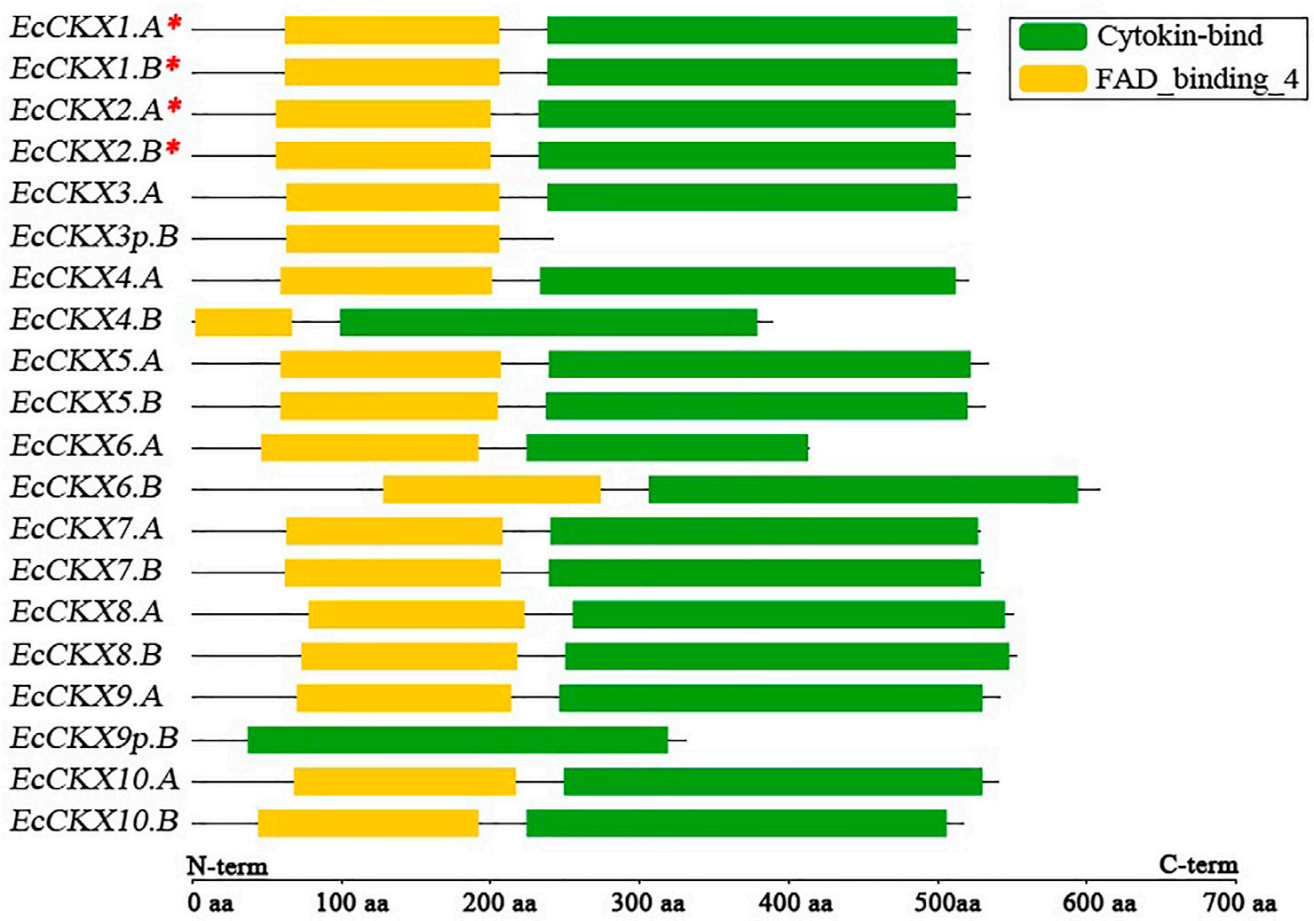
Putative motif/domain distribution in 20 translated peptide sequences, derived from identified *EcCKX* genes. A red asterix denotes genes identical to previously sequenced and characterized *EcCKX1* and *EcCKX2*.

Next, the exon-intron structure of the identified *EcCKX* (Figure 1), as well as the domain organization of the encoded EcCKX peptides were investigated (Figure 2). The number of exons was not conserved among members of the CKX gene family. Type I members, such as *EcCKX9.A* and *EcCKX8.A*/*B*, had three exons, while others, *EcCKX7.A*/*B* and *EcCKX10.A*/*B*, had only two exon structures, most likely due to the reduction of the intron2. On the other hand, most representatives of type II and III had five exons, with a few exceptions, such as *EcCKX6.A* and *EcCKX6.B*, which had 3- and 4-exon structures respectively. *EcCKX3p.B* and *EcCKX9p.B* genes, which showed a significant reduction in their length, lacked several exons in their structure. For example, *EcCKX3p.B* had only exon1 and exon2, while exons3-5 were eliminated. At the same time, exon1 was lost in *EcCKX9p.B* if compared to its homeolog, *EcCKX9.A*.

Typically, CKX proteins should contain two major domains (motifs) in their structure, which are responsible for the FAD- and CK-binding activity of the enzymes. Analysis of the translated peptides of the identified *EcCKX* revealed the presence and location of these crucial structural elements (Figure 2). Most of the identified genes contained both FAD- and CK-binding domains, despite the significant difference in the exon-intron structure of the genes. Usually, the FAD-binding domain is located above the CK-binding ones. Notably, *EcCKX6.B*, which had an enlarged exon1, also had a longer variable sequence, upstream to the FAD-binding domain, while the *EcCKX6.B* peptide had the longest sequence of 609 amino acids—almost 55 amino acids longer than the typical *EcCKX* protein, and 195 amino acids longer than the peptide of its homeolog *EcCKX6.A*.

It is also worth noting that *EcCKX1.A*/*B* and *EcCKX2.A*/*B*, orthologs of the previously-described *EcCKX1* and *EcCKX2*, had a conserved protein length of 523 amino acids and domain organization, despite the significant sequence differences that led to their classification into distinct CKX types. Most of the homeologous pairs of genes have preserved the identical domain organization of their peptides. However, such representatives as *EcCKX3p.B* lacked the CK-binding domain, which correlates well with the loss of exons3-5 of this gene. Similarly, the peptide of *EcCKX9p.B* lacked the FAD-binding domain, while the gene did not contain exon1, which should be present in a normal gene, e.g., in its homeolog. Based on these findings, it is hypothesized that *EcCKX3p.B* and *EcCKX9p.B* may be pseudogenes that potentially faced selective pressure after the allopolyploidization of the *Eleusine* species, leading to the origin of *E. coracana*.

Further, analyses were performed of the rate of non-synonymous substitution (K_A_), the rate of synonymous substitutions (K_S_), and their ratio (K_A_/K_S_) (Table 2). All of the *EcCKX* homeologous pairs showed extremely low K_A_ values—in a range of 0.005–0.036—while the rate of synonymous substitutions was higher: K_S_ = 0.019–0.142. Interestingly, *EcCKX2.A*/*B* possessed the lowest rates of substitutions (K_A_ = 0.005; K_S_ = 0.019) among all identified *EcCKX*. *EcCKX7.A*/*B* showed the highest rate of non-synonymous substitutions (K_A_ = 0.036), which led to the highest K_A_/K_S_ ratio—0.447. All homeologous pairs of *EcCKX* showed K_A_/K_S_ values lower than 1 (in a range of 0.155–0.447), meaning that all of these duplicated genes were under purifying or stabilizing selection. *EcCKX1. A*/*B* possessed the lowest value of K_A_/K_S_ at 0.155. Such low values of K_A_/K_S_ ratios among *EcCKX* homeologs may suggest that the duplicated genes will be further preserved in their current state, and not face loss of function or neofunctionalization.

**TABLE 2:** Rates of (non-)synonymous substitutions within homologous pairs of *EcCKX*.

Pair of homeologs	KA	KS	KA/KS
*EcCKX1.A/B*	0.010	0.066	0.155
*EcCKX2.A/B*	0.005	0.019	0.265
*EcCKX3.A/p.B* ^a^	0.022	0.056	0.399
*EcCKX4.A/B*	0.027	0.142	0.193
*EcCKX5.A/B*	0.009	0.047	0.180
*EcCKX6.A/B*	0.020	0.069	0.291
*EcCKX7.A/B*	0.036	0.081	0.447
*EcCKX8.A/B*	0.018	0.062	0.287
*EcCKX9.A/p.B* ^a^	0.025	0.104	0.235
*EcCKX10.A/B*	0.023	0.107	0.217

a One of the genes is a pseudogene.

### Organization of *cis*-elements in upstream regions of *EcCKX*

**FIGURE 3:**
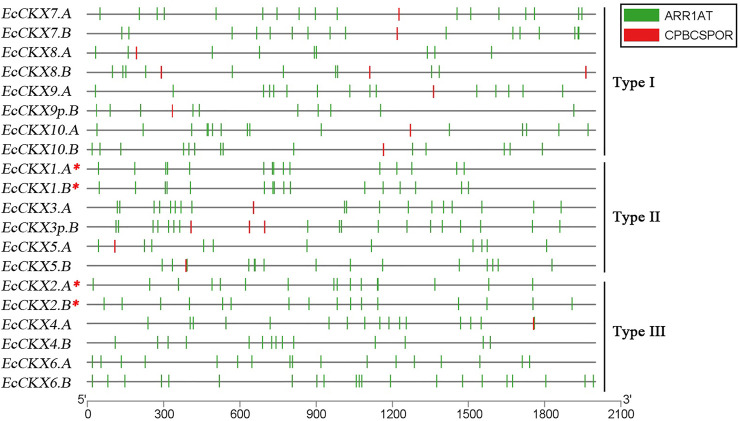
Presence of *cis*-elements, responsible for CK-mediated expression induction, in the 2 kb upstream region of the identified *EcCKX* genes of finger millet. A red asterix denotes genes identical to previously sequenced and characterized *EcCKX1* and *EcCKX2*.

To identify the putative *cis*-acting regulatory elements, the 2 kbp upstream sequences (from the start codon) were analyzed for all sets of the identified *EcCKX* (Figure 3). The upstream regions of various *EcCKX*s appear to be rich in various *cis*-regulatory elements, responsible for inducing expression in response to a wide range of factors. However, from the perspective of this study, the most interesting elements are those involved in cytokinin-mediated induction of expression. The PLACE database provides for analysis of only four known and confirmed *cis*-elements associated with CK-response: ARR1 (PLACE ID: S000454), AS1LIKECSHPRA (S000260), CPBCSPOR (S000491), and CYTOSITECSHPRA (S000260). Although the sequences of these *cis*-elements may vary depending on their type, all of them have a common tetranucleotide motif -NGAT- (more often -AGAT-). Only two types of *cis*-acting regulatory elements were identified in *EcCKX*: CPBCSPOR (-TATTAGN-, containing an inverted NGAT motif at 3′) and ARR1 (NGATT), with the latter being the most common. No AS1LIKECSHPRA or CYTOSITECSHPRA were detected.

On average, all identified *EcCKXs* contained 9–21 ARR1 and 1–3 CPBCSPOR elements in the 2 kbp upstream regions. It is interesting to note that type III members contained almost no CPBCSPOR elements in their upstream regions, except for *EcCKX4. A*, which had CPBCSPOR at position 1754–1759 bp, and overlapped another ARR1 element located at 1758–1762 bp. Often ARR1 elements were closely located, formed clusters (containing 2–3 ARR1 elements), or overlapped each other. However, the presence of two overlapping ARR1 (one straight and one inverted element), was identified in the upstream region of *EcCKX10.A* [463–473 (+) and 473–477 (-)], thus forming a -NGATTTAGN- palindromic sequence motif. For most of the CKX genes, the presence of a double placement of ARR1 elements in the upstream region (2–4 bp away from each other) was found, but their sequence had same direction, thus not forming palindromic motifs.

Different types of *CKX* genes had different combinations of regulatory *cis*-elements. For example, type I *EcCKX* had the lowest number of ARR1 elements, ranging from 9 to 17 ARR1 motifs per promoter region of each gene, whereas all type I genes, without exception, contained one to three CPBCSPOR motifs. For example, type I *EcCKX* had the lowest number of ARR1 elements, ranging from 9 to 17 ARR1 motifs per promoter region of each gene, whereas all type I genes contained from one to three CPBCSPOR motifs, without exception. In contrast, type III genes had the highest number of ARR1 regulatory elements in the upstream region (14–21 per gene), whereas CPBCSPOR motifs were absent. Only gene *EcCKX4.* had one CPBCSPOR element, which, however, overlapped the ARR1 motif sequence. Some of the genes had clusters of ARR1 motifs, where on a 100 bp sequence three or more ARR1 elements were present (e.g., *EcCKX6.B*).

Not all homeologous pairs shared the same patterns of ARR1 motif distribution. The structure of the upstream regions of *EcCKX1.A*/*B* was almost identical, while in the case of *EcCKX2.A*/*B*, it was similar only in a certain range—approx. 900–1,200 bp, etc. *EcCKX3p.B* displayed a very similar pattern of CK-responsive *cis*-elements to that of *EcCKX3.A*, whereas another possible pseudogene, *EcCKX9p.B*, had a distinct pattern of ARR1 allocation from its functional homeolog. Despite its pseudogenic nature, *EcCKX9p.B* had 10 ARR1, which is comparable to the upstream regions of other functional genes. It is unknown whether they were influenced by the process of gene disruption (possible loss of exon1 and its associated regions), or arose due to stochastic causes.

### Phylogenetic analysis of the cytokinin dehydrogenase/oxidase gene family

**FIGURE 4:**
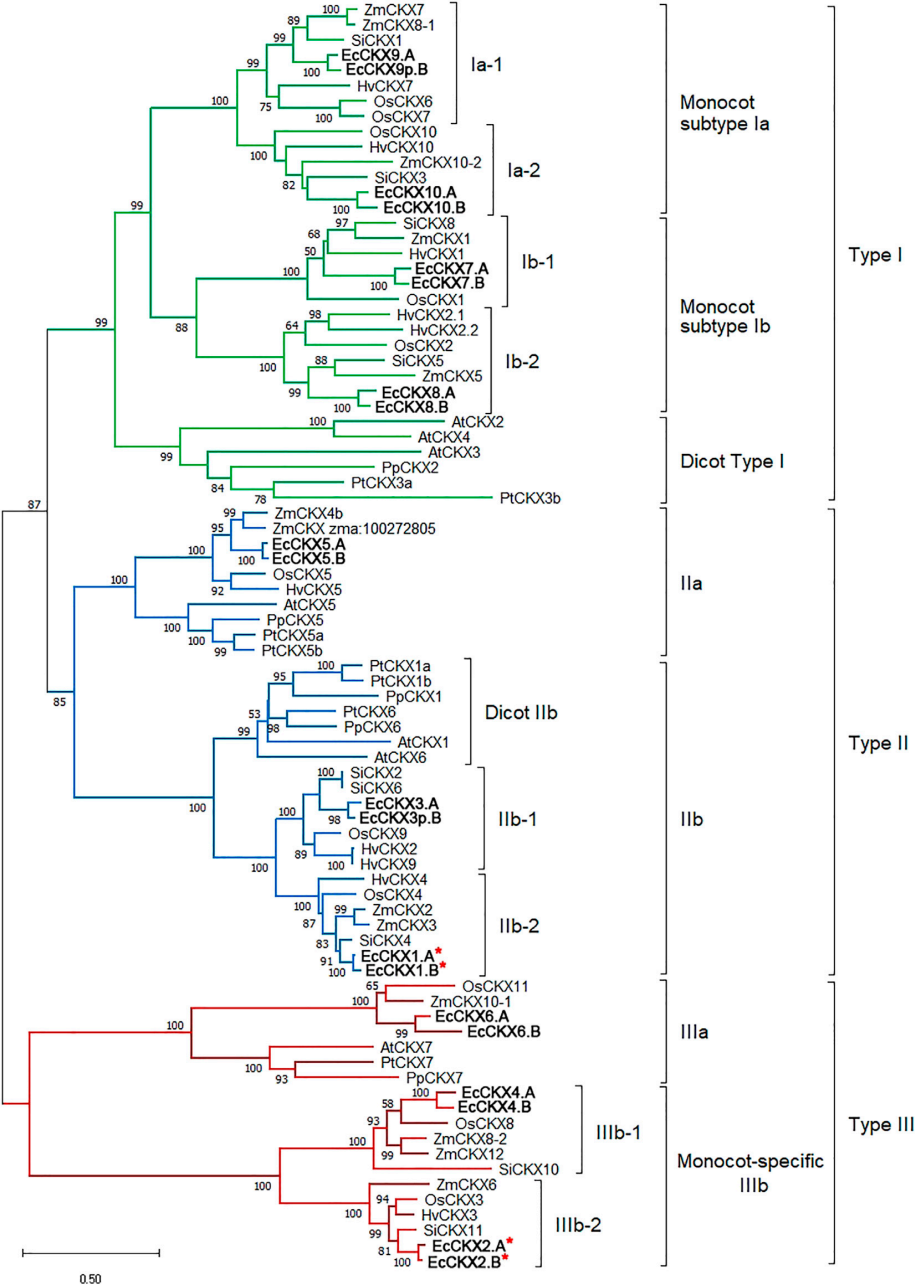
Unrooted ML phylogenetic tree for dicot (*A. thaliana*, *P. persica*, and *P. trichocarpa*) and monocot (*O. sativa*, *Z. mays*, *H. vulgare*, and *S. italica*) CKX proteins, as well as translated peptides of identified genes in *E. coracana* genome. The tree was constructed with bootstrap support of 1,000 iterations (50% and higher values are displayed). A red asterix denotes genes identical to previously sequenced and characterized *EcCKX1* and *EcCKX2*.

The next step was to analyze the phylogenic relationships between the identified *EcCKX* and members of this gene family in other species (Figure 4). In order to perform this analysis, 83 CKX amino acid sequences were aligned, and further phylogenic analysis was performed using the maximum likelihood (ML) method, which allowed us to obtain a tree with high confidence at all crucial branching nodes (bootstrap values for main nodes were 85 and higher). The results allowed us to conclude that CKX proteins can be divided into three main types, which include sequences of both monocotyledonous and dicotyledonous species. Seven functional EcCKX proteins and one reduced (EcCKX9p.B) were associated with type I CKX. Among the type I proteins, the vast majority came from monocot species, in which type I panel proteins expanded rapidly compared to dicot species. Dicot proteins (e.g., AtCKX2, AtCKX3, and AtCKX4) formed a separate branch of CKX, specific only to this taxonomic group (named Dicot type I). At the same time, monocot CKX proteins formed four minor clades, which could be grouped into subtype Ia of monocots belonging to EcCKX9.A, EcCKX10.A/B, and subtype Ib of monocots containing EcCKX7.A/B and EcCKX8.A/B.

Proteins of each minor clade Ia-1, Ia-2, Ib-1, and Ib-2, represent groups of sub-orthologs, as each clade contains the respective proteins from the analyzed monocot species: *O. sativa*, *Z. mays*, *S. italica*, *H. vulgare,* and *E. coracana*. This allows us to assume that diversification of these minor subtypes of CKX in monocots occurred at least before the emergence of the Poaceae species. Such diversity could potentially result from local (in the case of *EcCKX7.A/B* and *EcCKX8.A/B*) or genome-wide duplication.

Type II of CKX is comprised of two major subtypes: IIa and IIb, which can be further divided into three minor clades of Dicot-specific IIb, and Monocot IIb-1 and IIb-2. Similar to Type I, the diversity of the monocot CKX was higher than in the dicot. However, in the case of Type 2 CKX, dicot proteins did not group distinctly, but were present in subtype IIa (AtCKX5), IIb (AtCKX1, and AtCKX6) subtype clades. This indicates that *EcCKX5.A*/*B* appears to be an ancient paralog in relation to representatives of the IIb subtype (*EcCKX1.A*/*B* and *EcCKX3.A*), if the close allocation of *EcCKX5.A*/*B* and *EcCKX1.A*/*B* on the same chromosome is taken into account.

Proteins of Type III were separated into two branches. The primary branch (IIIa) included several monocot proteins and their orthologs from dicots (e.g., AtCKX7). *EcCKX6.A/B* were also attributed to this subtype. The second branch, subtype IIIb, contained only monocot CKX, and formed two minor clades representing distinct lineages of CKX peptide paralogs: IIIb-1 and IIIb-2, which included *EcCKX4.A/B* and *EcCKX2.A/B*, respectively. The previously-identified *EcCKX1* and *EcCKX2* were attributed to different types of CKX, despite their similarity in protein length, genomic structure, and potentially similar function. It is also worth noting that the branches within the IIb and IIIb clades were the shortest observed, compared to other subtypes of CKX, suggesting that *EcCKX1* and *EcCKX2* orthologs are conserved in monocots (at least among the Poaceae species). However, the fact that these genes were not eliminated, despite their paralogous nature, may indicate that multiple copies of particular *EcCKX* have undergone sub-functionalization, or that an increased number of their copies confer a selective advantage.

### Synteny of *EcCKX*

**FIGURE 5:**
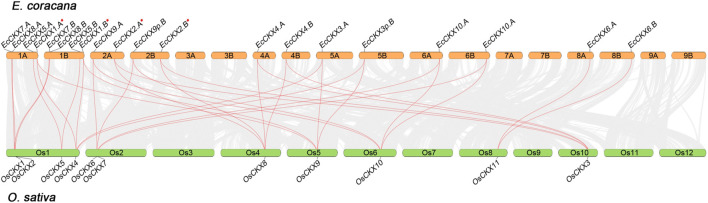
Synteny analysis of *CKX* genes between *E. coracana* and *O. sativa* species. The collinear blocks between *O. sativa* and *E. coracana* species are shown with gray lines, while syntenic pairs of *CKX* genes are highlighted in red. The chromosome number is indicated above each respective chromosome. A red asterix denotes genes identical to previously sequenced and characterized *EcCKX1* and *EcCKX2*.

**FIGURE 6:**
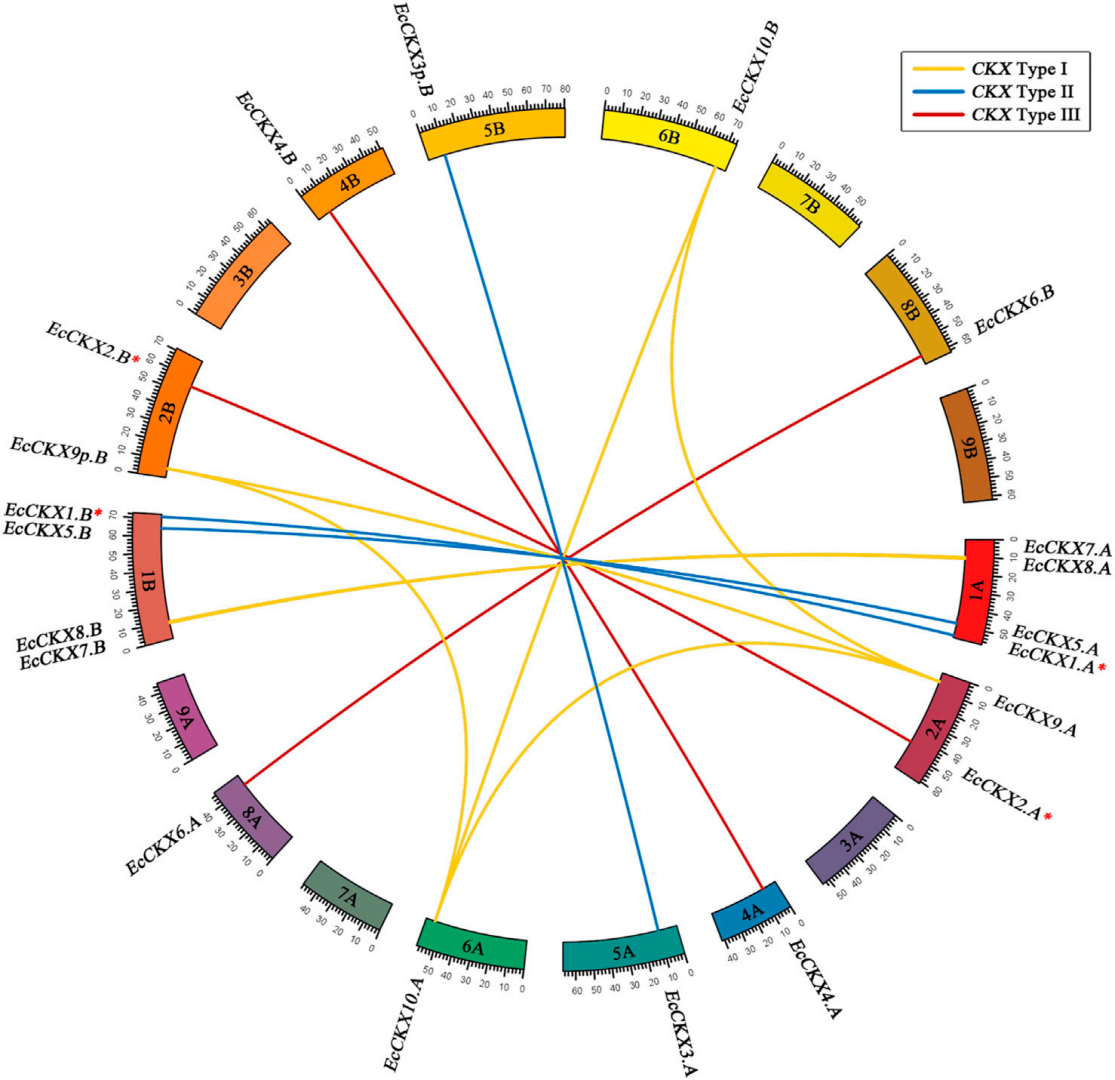
Synteny analysis of interchromosomal relationships of *EcCKX* of various types from different subgenomes of *E. coracana*. Syntenic gene pairs are colored according to the type of CKX, to which they are attributed. A red asterix denotes genes identical to previously sequenced and characterized *EcCKX1* and *EcCKX2*.

Furthermore, interchromosomal synteny of finger millet *CKX* was inferred (Figures 5, 6), which is of additional interest, since *E. coracana* is considered an allotetraploid species and has an expanded panel of *EcCKX*. Figure 6 shows the syntenic relations between the identified *EcCKX* genes. Thus, all the genes formed syntenic pairs with their homeologs from A and B subgenomes, which further confirms the hypothesis that the rapid expansion of the *CKX* gene family in *E. coracana* is the result of an allopolyploidization event. Only genes from Type I formed pairs, not only between homeologs, but also with paralogs of the same type. For example. *EcCKX10.A*/*B* formed syntenic pairs with *EcCKX9.A*. This suggests that the sequences of these genes remained sufficiently conserved, and that they were recognized as possible duplicates during the synteny analysis. No syntenic pairs between genes of different CKX isotypes of Type II or III were detected.

Intergenomic synteny between *E. coracana* and the model crop species *O. sativa* made it possible to identify orthologs of *EcCKX* (Figure 5). The potential duplicates *EcCKX7.A*/*B* and *EcCKX8A*/*B* share the same loci on chromosomes 1A and 1B of finger millet, similar to their orthologs *OsCKX1* and *OsCKX2* in the rice genome. At the same time, the orthologs of *EcCKX1.A*/*B* and *EcCKX5.A*/*B*—*OsCKX5* and *OsCKX4*—were located distantly on Os1 in the rice genome and formed syntenic pairs with only one of their direct orthologs in *E. coracana*. Taking into account the significant phylogenetic distance between the *EcCKX1.A*/*B*-*OsCKX5* and *EcCKX5.A*/*B*-*OsCKX4* clades, it may be concluded that the lineages of these genes did not arise as a result of a recent duplication event.

At the same time, members of Type III, *EcCKX2.A*/*B* and *EcCKX4.A*/*B*, each formed syntenic pairs with both of their orthologs in rice: *OsCKX3* and *OsCKX8*. Unlike the previous case, this finding confirms the paralogous nature of the relatively recent duplicates *EcCKX2* and *EcCKX4*. Similarly, members of Type III, *EcCKX9.A*/*B* and *EcCKX10.A*/*B*, formed double pairs with *OsCKX6* and *OsCKX7*. It is worth noting that *OsCKX6* and *OsCKX7* are located relatively close on the Os2 chromosome, which may indicate the origin of these paralogs from a tandem duplication event.

Apart from the other genes, *EcCKX6.A*/*B* formed a syntenic pair only with *OsCKX11*. Both of these genes belong to the sub-Type IIIa (Figure 5), the clade which also contains dicot orthologs. This confirms that the present gene of this CKX isotype was evolutionarily preserved and diversified from other Type III genes, at least in the last common ancestor of dicots and monocots.

### Expression profiling of *EcCKX*

**FIGURE 7:**
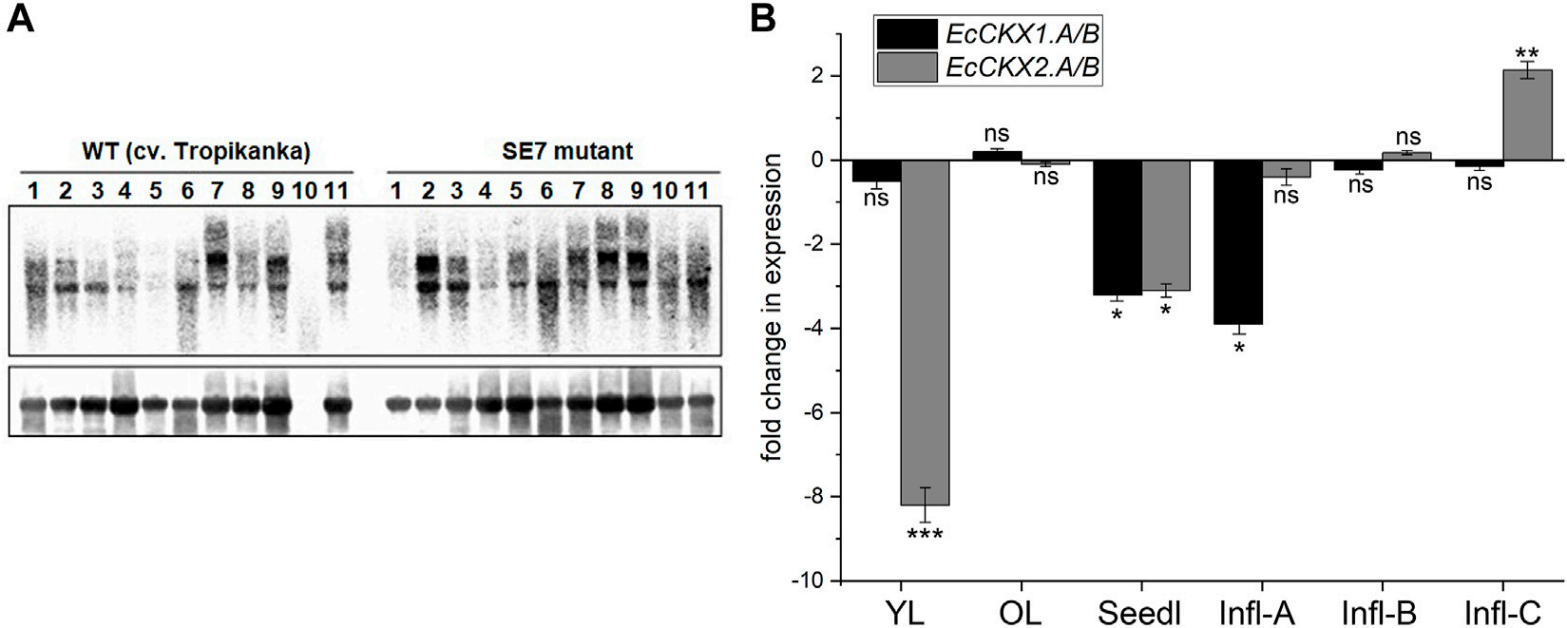
Expression profiling of *EcCKX* genes in the *E. coracana* mutant SE7 line. **(A)**—Northern blot of different tissues and developing stages of wild type and mutant SE7 finger millet plants hybridized with the *EcCKX* sequence as a probe (above): 1—node; 2—upper leaf; 3—lower leaf; 4—internode; 5—young root; 6—seedling 4 DAI; 7—developing panicle, < 1 cm in length; 8—developing panicle, 2–3 cm; 9—developing panicle, 3–4 cm; 10—developing panicle, > 4 cm; 11—seedling, 5 DAI. The signals were quantified by sequential hybridization with a ribosomal fragment as probe (below). **(B)**—Transcript profiling of the *EcCKX1. A*/*B* and *EcCKX2. A*/*B* genes in the developing inflorescences and young leaves of finger millet determined by quantitative RT-PCR analysis: YL—young leaf; OL—old leaf; Seedl—seedling 5 DAI; Infl-A/B/C—inflorescence tissue at stage A, B, and C. Data shown as fold change of upregulation of downregulation of respective gene in SE7 mutant line, compared to WT: ns—difference not statistically significant, *—significant at *p* < 0.05; **—*p* < 0.01; ***—*p* < 0.001.

Using the previously confirmed *CKX*-deficient lines of *E. coracana* somaclonal mutants (Radchuk et al., 2012; Bayer et al., 2014), we performed an expression profiling of *EcCKX* and *EcCKX1.A*/*B* genes, in particular of *EcCKX2.A*/*B* (Figure 7). As mentioned above, homeologous pairs of *EcCKX1.A*/*B* and *EcCKX2.A*/*B* encode highly conserved peptide products with sequence similarities up to 97.7% and 98.85%, respectively. It is the highest degree of conservation among all identified *EcCKX* homeolog pairs. Since the transcripts of these genes are hard to distinguish and are highly conserved, the expression of *EcCKX1.A*/*B* and *EcCKX2.A*/*B* was analyzed jointly for both homologous copies (Figure 7B).

The northern blot array allowed us to determine the total level of *EcCKX* transcripts in different *E. coracana* tissues (Figure 7A). For example, the upper leaves of the SE7 line were found to have higher levels of *EcCKX* transcripts than the WT line. Similarly, young roots of SE7 plants had higher levels of *EcCKX* transcripts compared to the WT, while no significant differences in *EcCKX* levels were found at 4 and 5 DAI in either line. However, at early stages of panicle development, SE7 plants had a lower level of *EcCKX* expression than the WT, whereas at later stages, the total levels of expression were comparable, or the *EcCKX* expression in SE7 could even be higher. The total *EcCKX* expression in the nodes and internodes of SE7 was lower than in the WT.

Previously, total transcriptomic analysis revealed the genes responsible for the semi-dwarf and bushy phenotype of SE7 (Radchuk et al., 2012). In this study, a detailed analysis of the expression of *EcCKX1.A*/*B* and *EcCKX2.A*/*B* reveals patterns of changes in the expression of these genes in the SE7 mutant, especially given that the SE7 has normal rates of CK synthesis (Radchuk et al., 2012). The most dramatic decrease in *EcCKX2.A*/*B* expression is observed in young leaves of SE7 (more than 8-fold), compared to the wild type, whereas the levels of *EcCKX1.A*/*B* remain unchanged (Figure 7B). At the same time, these genes exhibit no significant changes in expression in old leaves. However, in seedlings (5 DAI), the expression of both *EcCKX1.A*/*B* and *EcCKX2.A*/*B* decreases 3.1 to 3.2-fold, whereas analysis of total *EcCKX* expression shows no significant difference. It is possible that during germination the total level of these CK-degrading enzymes remains high, thus partially compensating for the deficiency of the *EcCKX1.A*/*B* and *EcCKX2.A*/*B* isotypes, possibly through the increased expression of their paralogs.

Most interestingly, *EcCKX1.A*/*B* expression levels change during early panicle development, while no differences in *EcCKX2.A*/*B* expression are found in the SE7 and WT lines. The transcript levels of *EcCKX1.A*/*B* are found to be nearly 3.9-fold lower in SE7 inflorescences during the early inflorescence development (stage A). It is noteworthy that at later stages the expression of *EcCKX1.A*/*B,* as well as that of *EcCKX2.A*/*B,* remains unchanged. However, in the later stage of inflorescence development (stage C), *EcCKX2.A*/*B* shows a 2.13-fold increase in expression. This correlates well with the observed increase in the total *EcCKX* level of transcripts at late stages of inflorescence development. Perhaps this observation can serve as a partial confirmation of the stage-dependent specificity of the expression of these two genes. Most likely, the early stages of panicle growth, such as stage A, are the key phases, in which high levels of CK determine subsequent panicle/inflorescence size, thus shaping crop grain productivity.

### Phenotypic changes in cytokinin dehydrogenase/oxidase-deficient somaclonal mutants of *E. coracana*

**FIGURE 8:**
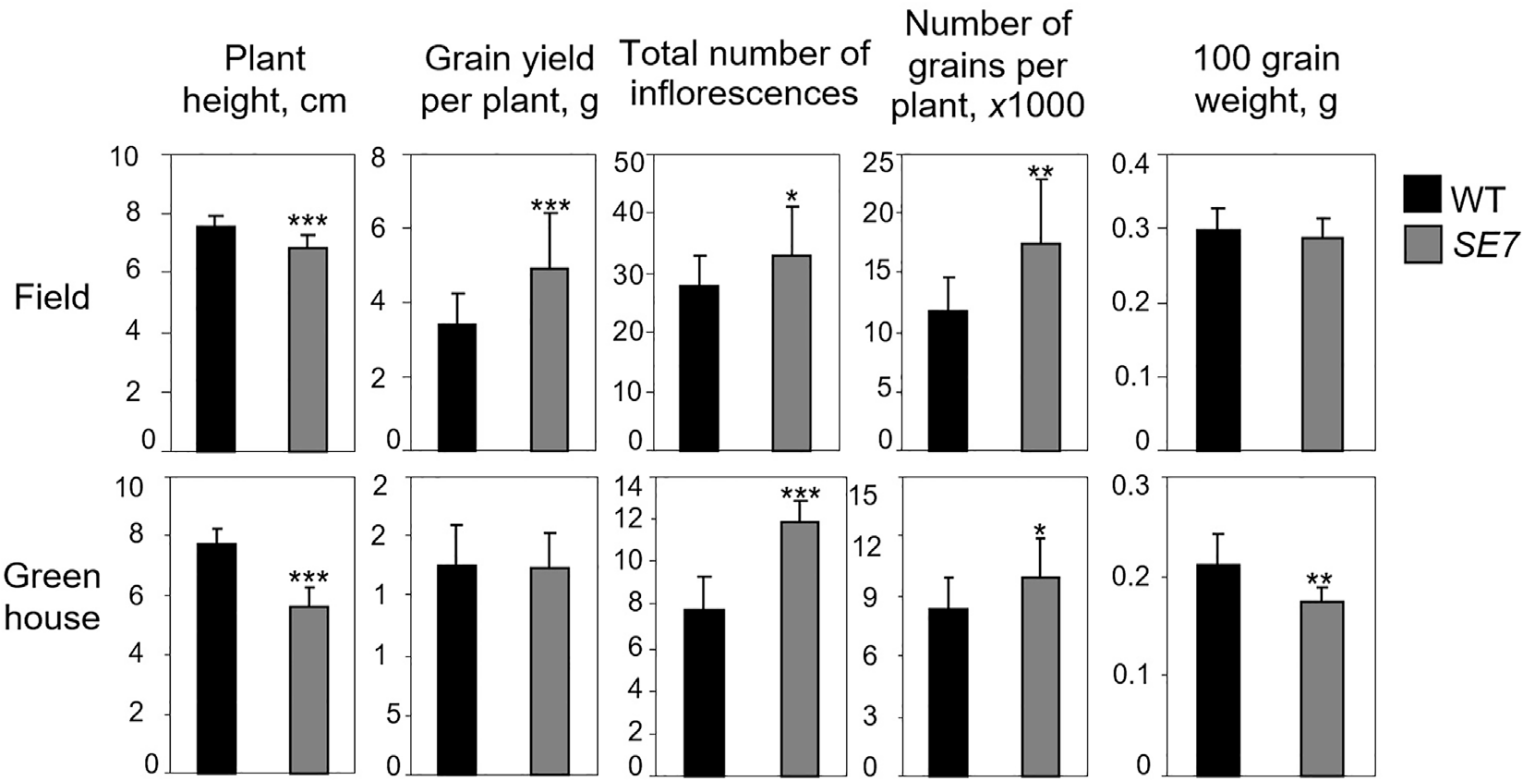
Productivity trait difference of field-grown (upper panel) and greenhouse-grown (lower panel) SE7 somaclonal mutant plants compared to the wild type of finger millet. Statistically significant differences are marked with *—significant at *p* < 0.05; **—*p* < 0.01; ***—*p* < 0.001.

For field evaluation of the phenotype, around 100 wild type and SE7 finger millet plants were grown for 2 weeks in a greenhouse and then transplanted in May to a field in Gatersleben, Germany, using 20 cm plant spacing. Plant height and the yields from 25 plants were measured in late September. For greenhouse phenotype evaluation, 25 SE7 mutant and wild-type plants were grown in a greenhouse during the same period (Figure 8).

SE7 somatic mutant plants grown in both the field and the greenhouse were characterized by significantly lower plant height (Figure 8) than the wild type, whereas greenhouse-grown plants differed more. The total number of inflorescences per plant also increased, indicating that more vegetative meristems were formed in SE7 plants (Figure 8). Surprisingly, greenhouse-grown plants did not show any significant difference in this parameter, probably due to altered growing conditions. Also, the *CKX*-deficient mutant line had a shortened vegetation cycle.

The mutant plants also produced more seeds per plant compared to the wild type, indicating extensive production of generative meristems in the SE7 line in response to higher levels of CK, which were not degraded (Figure 8). Increased numbers of inflorescences and flowers led to a significantly higher grain yield per plant in field conditions. However, this effect was less significant among the greenhouse-grown plants, since under such conditions, both lines produced more inflorescences, and thus showed higher grain productivity (Figure 8). The less significant difference in total plant productivity in the greenhouse could be explained by the fact that greenhouse-grown SE7 plants had a significantly lower grain mass. In contrast, 100-grain weight was not significantly different between field-grown SE7 and WT plants. It can therefore be concluded that the increase in the number of inflorescences is a key factor in determining the increased grain productivity (per plant) of the SE7 line (Figure 8).

**FIGURE 9:**
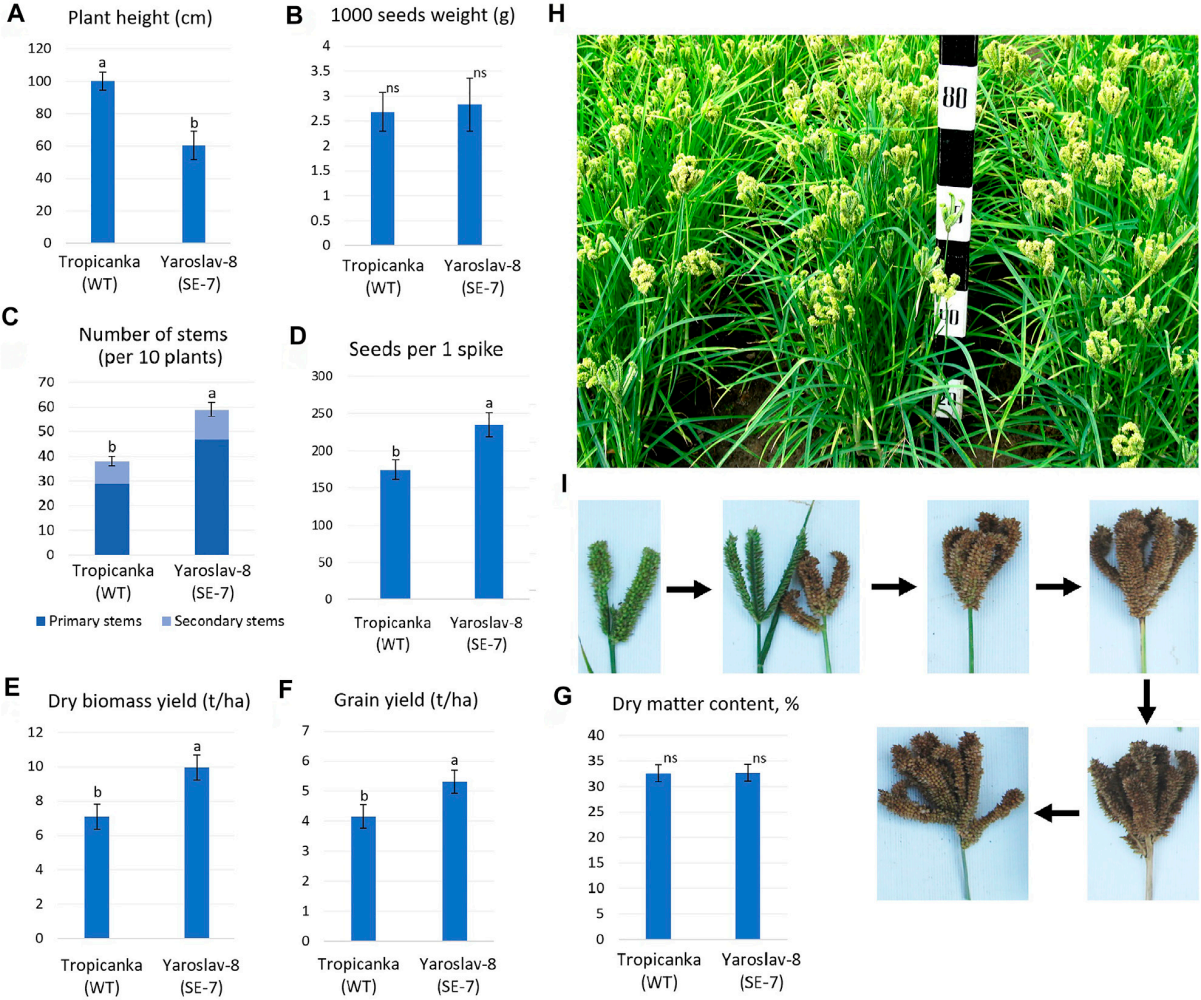
Performance of the SE7 mutant line in comparison with the WT during field trials. **(A–D)**, **(G)**—measurements of different productivity traits; **(E,F)**—grain and biomass yield; **(H)**— plants of the SE7 line during maturation; **(I)**—different stages of finger millet panicle maturation. Different letters within each parameter **(A–G)**: a, b—significantly different values; ns—difference not significant (at *p* < 0.05, Fischer LSD).

Following the successful comparison of SE7 plant performance, and confirmation that the increased plant productivity is also found in field-grown plants, a full-scale field trial was conducted using the SE7 line and its WT (Figure 9). The most prominent features of the obtained somaclonal line SE7 were significantly reduced height, compared to the parental genotype cv. Tropikanka, and a much greater bush density, due to an increased number of primary and lateral stems. However, the somaclones did not show the reduced grain size. It was established that the SE7 line had a reduced height of almost 40%, compared to the WT (60.4±8.8 v. 100.1±5.5 cm, Figure 9A). Similar to the comparison shown in Figure 8, the weight of 1,000 grains manifest no significant change during field trials (Figure 9B).

At the same time, the SE7 had a significantly greater number of primary stems (48±2.9 v. 29±1.9 in WT), while the number of secondary stems was similar for both the SE7 and WT—nine on average. The number of grains per spike increased by 34.3% in the SE7 line (236±16 vs. 175±13), compared to the WT (Figures 9C,D). From the concluding observations of two independent studies, it can be summarized that the key factors shaping the increased productivity of the *CKX*-deficient line are an increase in the number of inflorescences (due to the larger number of primary stems), and an increase in the number of seeds per panicle, with grain weight not significantly affecting the resulting plant productivity.

The resulting grain productivity of the SE7 line reached 5,320 kg/ha on average, 27.9% higher than the WT, which produced 4,159 kg/ha of grain (Figure 9F). In addition, the SE7 line showed increased levels of biomass accumulation, which is associated with the previously-identified downregulation of *CKX*. The SE7 line showed almost 10 t/ha of straw (absolutely dry mass), which is higher than that of the WT at 7.1 t/ha. Such a substantial increase in biomass productivity is most likely associated with the appearance of additional stems.

## Discussion

A total of 20 *EcCKX*s were identified in the latest *E. coracana* assembly. Two of all the identified genes are considered pseudogenes, as they lack important structural domains: FAD- and CK-binding domains. The rapid expansion of the CKX gene family in finger millet can be associated with the evolutionary history of this species, which includes at least one allopolyploidization event giving rise to modern *E. coracana* (Zhang et al., 2019a; 2019b). *EcCKX* *s* are represented by complete homeologous pairs, indicating that the majority of these duplicated genes were not eliminated, but most likely passed through subfunctionalization. Using the example of *Brassica rapa,* it is shown that *CKX*, resulting from segmental duplications, tend to change their expression patterns and thus potentially gain new functions (Liu et al., 2013). It is obvious that in such a case, the duplicated genes are not eliminated or pseudogenized over time.

Other species closely related to *E. coracana* contain a lower number of *CKX* in their genomes. For example, *S. italica* has only 11 *CKX* genes (Wang et al., 2014); *H. vulgare* has 13–14 genes (Zalewski et al., 2014); *O. sativa* has 11; *Brachypodium distachyon* has 13; *Sorghum bicolor* has 12; and *Z. mays* has 16 (Mameaux et al., 2012). Genomes of dicot species can contain 7 *CKX* genes, as is observed in *A. thaliana*, while some species can contain a lower number of *CKX* genes, such as *P. persica* which has only 6; or more, e.g., 8 genes in *P. trichocarpa* (Immanen et al., 2013). Polyploid dicots possess much larger panels of *CKX* genes, for example *Glycine max* has 16 genes (Liu et al., 2021), while polyploid *Brassica* species have a much larger gene family compared to diploids. While *B. rapa* has only 12 *CKX* genes (Liu et al., 2013), amphidiploid *B. napus* contains 23 (Liu et al., 2018), while *B. oleracea* accounts for up to 36 *CKX* genes (Zhu et al., 2022).

Analysis of *cis*-regulatory elements make it possible to reveal the patterns of distribution of CK-responsive elements within the identified genes. Understanding of the diversity of CK-responsive *cis*-elements is currently limited to only a few validated motifs, such as ARR1 (AGAT [T,C] motif) (Brenner and Schmülling, 2015). However, it is evident that the expression of *CKX* is specifically induced by CK (Curaba et al., 2014), hence, new types of CK-responsive *cis*-elements have yet to be described. In this study we find unconventional motif organization, such as the presence of palindromic ARR1s or overlapping inverted ARR1-CPBCSPOR motifs. However, it is important to note that *in silico* identification of *cis*-regulatory elements, especially short-sequence motifs (such as ARR1, discussed above), is not enough for prediction of the binding site of functional proteins involved in transcriptional regulation (Hernandez-Garcia and Finer, 2014). Despite the presence of numerous copies of short-sequence motifs in the genome, only a small part of them are accessible for protein binding and further regulation of gene expression (Hardison and Taylor, 2012). Similarly, as analyzed in our study, ARR1 elements consist of 3–4 nucleotides. It may be expected that only a few of the identified *cis*-elements might be involved in CK-mediated expression of *EcCKX*. This could be a question for further separate research.

It is not clear what the role of such a broad diversity of these genes is in different taxa. However, the so-called “ancient” *CKXs* (which have direct orthologs in non-angiosperm species) have recently been found to preferentially degrade *cis*-zeatin (*c*Z)-type cytokinins, while non-ancient (relatively recent duplicates) mostly target *N* ^6^-(Δ^2^-isopentenyl) adenines (iPs) and *trans*-zeatins (tZs) (Wang et al., 2021). In addition, it has been hypothesized that non-ancient CKX and their substrates (iP) may regulate the development of plant organs, especially flowers, and may regulate stress responses (Wang et al., 2021). Rapidly expanded type I *CKXs* belong to such non-ancient type genes, since their entire group is sub-orthologous to *AtCKX4*, which is considered one of the remarkable representatives of recent *CKXs*. Similarly, groups IIa, IIb, and IIIb are also orthologous to such non-ancient *CKX*, including *EcCKX1.A*/*B* and *EcCKX2.A*/*B* (widely discussed in this study), the deficiency of which results in increased inflorescence/panicle. By contrast, the phylogenetic group IIIa is orthologous with *AtCKX7*—the gene of ancient-type *CKX*. Genes *EcCKX6.A*/*B* belong to this group. Notably, this *CKX* homeolog pair has very strict orthology with *OsCKX11* of rice, unlike other *EcCKX*. In a detailed study on Poaceae *CKX*, a clade containing *OsCKX11* orthologs, clustered as basal to all monocot *CKXs* (Mameaux et al., 2012), further supports the assignment to “ancient” type genes.

Measurement of CK species in finger millet identifies iP as the major form of CKs in developing inflorescences of this species (Radchuk et al., 2012). When we analyzed the somaclonal variant *SE7*, produced previously (Baer et al., 2007), the levels of iP and the iP precursor riboside phosphate were strongly increased early in inflorescences of this genotype, but did not change compared to the wild type later on (Radchuk et al., 2012). We hypothesized that increased iP levels are probably due to impaired degradation, as evidenced by lower *EcCKX* expression in young inflorescences. Such disturbed CK homeostasis, brought about by either reduced CK degradation or biosynthesis, can stimulate plant productivity. As observed in the present study, effects of *EcCKX* deficiency in the SE7 mutant may contribute to an understanding of the phenotypic effects caused by *CKX* downregulation. Analyzed *EcCKX1.A*/*B* and *EcCKX2.A*/*B* were preferentially down-regulated in actively growing and generative tissues, leading to rapid increase of grain and biomass productivity of the mutant. Increased plant productivity of the SE7 line may be beneficial not only for food production, but may also open ways to use finger millet for biofuel production, in particular, the production of second-generation bioethanol (Yemets et al., 2020; Blume et al., 2021).

Initially, down-regulated *CKX* expression was shown to decrease CK-degradation activity and increase CK levels in rice, leading to an increase of seed numbers per plant and 1000-grain weight (Ashikari et al., 2005). The same evidence was then obtained in experiments with RNAi-based silencing of the *HvCKX1* in barley, resulting in reduced cytokinin oxidase/dehydrogenase levels and higher plant productivity (Zalewski et al., 2010). This is confirmed in other publications by Zalewski et al. (2012; 2014).

Later, *HvCKX1* and *HvCKX9*, predominantly expressed in the aleurone layer of maturing grains and leaf vasculature, were used for stable *Agrobacterium tumefaciens*-mediated transformation of the barley cultivar Golden Promise (Mrízová et al., 2013). As a result, constitutive overexpression of these two genes independently induced morphological changes in barley plants and prevented their transition to flowering. In all obtained transgenic lines, roots proliferated more rapidly and root-to-shoot ratios were higher than in wild-type plants. Depleted CK levels during early phases of development are restored by down-regulation of endogenous *CKX* and reinforced *de novo* biosynthesis of CKs. When barley plants were transformed with *Arabidopsis AtCKX1* under a mild root-preferred promoter, the obtained transgenic lines were found to have higher drought tolerance then the control (Pospíšilová et al., 2016). These transgenic plants can maintain higher yield parameters following revitalization compared to wild type plants under stress conditions. Knockdown of the inflorescence meristem-specific cytokinin oxidase in rice (*OsCKX2*) regulates primary flowering activity, and modulates rice grain yield under normal conditions as well as under abiotic stress conditions by controlling cytokinin levels (Joshi et al., 2018).

Several recent studies have demonstrated that RNAi-based silencing of *CKX* leads to increased grain yields in other cereals. In particular, a significantly increased grain number per spike was found as the effect of *TaCKX2.4* silencing by RNAi in wheat (Li et al., 2018). Coordinated effects of *TaCKX1* silencing on the expression of other *TaCKX*, wheat spike phytohormone levels, and yield-related traits were demonstrated in silenced T2 lines (Jabłoński et al., 2020). In the moss *Physcomitrella patens*, an evolutionarily early divergent plant, it was shown that under normal growth conditions overexpression of *PpCKX1* caused many phenotypic changes at different developmental stages, and that increased growth of the rhizoid could affect these changes. In addition, evidence is provided that the *PpCKX1*-overexpressing plants show enhanced dehydration and salt stress tolerance. Taken together, the authors suggested that *PpCKX1* plays a regulatory role in the development and adaptation to abiotic stresses of this evolutionarily early land plant species (Hyoung et al., 2020).

Finally, knockout mutation of *HvCKX1* by CRISPR/Cas9 editing in barley had a limited effect on yield productivity. However, significant reductions in CKX enzyme activity in young spikelets and 10-day-old roots corresponded to greater root length, number of root hairs, and increased surface area (Gasparis et al., 2019). In contrast, the roots of *ckx3* knockout mutants were smaller. The observed changes suggest that the knockout of a single *CKX* in barley may not be sufficient to disrupt cytokinin homeostasis or increase grain yields. These authors then demonstrated that underlying mechanisms of co-regulation of the expression of *TaCKX* family members were similar in different spring wheat cultivars, but, depending on the content and composition of phytohormones, regulation of yield-related traits was differentially affected (Jablonski et al., 2021). On the other hand, identification and characterization of all members of the *CKX* family, not only in *E. coracana*, but in closely-related species with already characterized genomes and transcriptomes (Zhang et al., 2019a; Zhang et al., 2019b; Hall et al., 2021), may provide more information about functional features of different *CKX* types.

Successful application of genome editing technics, targeting *CKX* genes in different taxa, will require a comprehensive understanding of genomic organization, phylogenetic classification, and evolution of *CKX* genes, in order to efficiently affect all paralogous copies with similar function and achieve desirable effects of genetic engineering. The greater diversity of *CKX* in monocots compared to dicots, and the relationship between different metabolic pathways of different forms of cytokinins, remains unclear. Understanding of these matters may become an important tool for targeted crop design to ensure global food security in the future.

## Data Availability

The original contributions presented in the study are included in the article/Supplementary Material. Further inquiries can be directed to the corresponding author.
