# Supplementary material for: Genome-wide identification and analysis of the cytokinin oxidase/dehydrogenase (ckx) gene family in finger millet (Eleusine coracana)
Source: Front Genet. 2022 Sep 27;13:963789. doi: 10.3389/fgene.2022.963789 (PMC9589517; doi:10.3389/fgene.2022.963789)
Supplement: Supplementary file 1 [file DataSheet1.DOCX]

**Supplementary file 1**

**Supplementary Table 1.** List of CKX proteins, used for the ML phylogenetic tree construction

| Species | Protein name* | Protein ID (Uniprot, NCBI or Phytozome ID) |
| --- | --- | --- |
| *Arabidopsis thaliana* | AtCKX1 | NP_001318403 |
|  | AtCKX2 | NP_565455 |
|  | AtCKX3 | NP_200507 |
|  | AtCKX4 | NP_194703 |
|  | AtCKX5 | NP_177678 |
|  | AtCKX6 | NP_191903 |
|  | AtCKX7 | NP_850863 |
| *Prunus* *persica* | PpCKX1 | Prupe.7G052300 |
|  | PpCKX2 | Prupe.7G208500 |
|  | PpCKX5 | Prupe.1G373300 |
|  | PpCKX6 | Prupe.2G026700 |
|  | PpCKX7 | Prupe.1G404300 |
| *Populus trichocarpa* | PtCKX1a | Potri.006G047900 |
|  | PtCKX1b | Potri.016G044100 |
|  | PtCKX3a | Potri.006G152500 |
|  | PtCKX3b | Potri.007G066100 |
|  | PtCKX5a | Potri.002G030500 |
|  | PtCKX5b | Potri.005G232300 |
|  | PtCKX6 | Potri.003G203600 |
|  | PtCKX7 | Potri.006G221000 |
| *Setaria italica* | SiCKX1 | Si019278m.g |
|  | SiCKX2 | Si021761m.g |
|  | SiCKX3 | Si008391m.g |
|  | SiCKX4 | Si001018m.g |
|  | SiCKX5 | Si001500m.g |
|  | SiCKX6 | Si000960m.g |
|  | SiCKX8 | Si001019m.g |
|  | SiCKX10 | Si012051m.g |
|  | SiCKX11 | Si035175m.g |
| *Oryza sativa* | OsCKX1 | XP_015635851 |
|  | OsCKX2 | XP_015629416 |
|  | OsCKX3 | XP_015613042 |
|  | OsCKX4 | XP_015621343 |
|  | OsCKX5 | XP_015625924 |
|  | OsCKX6 | XP_015624918 |
|  | OsCKX7 | XP_015624361 |
|  | OsCKX8 | XP_015633517 |
|  | OsCKX9 | XP_015639136 |
|  | OsCKX10 | XP_015642364 |
|  | OsCKX11 | XP_015650661 |

**Table S1.** Continued…

| Species | Protein name | Protein ID (Uniprot, NCBI or Phytozome ID) |
| --- | --- | --- |
| *Zea mays* | ZmCKX1 | NP_001105591 |
|  | ZmCKX2 | NP_001105526 |
|  | ZmCKX3 | NP_001105163 |
|  | ZmCKX4b | NP_001185960 |
|  | ZmCKX5 | NP_001185958 |
|  | ZmCKX6 | NP_001185959 |
|  | ZmCKX7 | XP_008679837 |
|  | ZmCKX8-1 | NP_001185809 |
|  | ZmCKX8-2 | XP_008663392 |
|  | ZmCKX10-1 | NP_001146838 |
|  | ZmCKX10-2 | XP_008660924 |
|  | ZmCKX12 | NP_001185961 |
|  | ZmCKX_zma:  100272805 | NP_001347058 |
| *Hordeum vulgare* | HvCKX1 | JF495479 |
|  | HvCKX2 | AF490591 |
|  | HvCKX2.1 | JF495488 |
|  | HvCKX2.2 | JF495489 |
|  | HvCKX3 | JF495480 |
|  | HvCKX4 | JF495481 |
|  | HvCKX5 | AK370106 |
|  | HvCKX7 | JF495483 |
|  | HvCKX9 | JF495484 |
|  | HvCKX10 | JF495485 |
| *Eleusine coracana* | For IDs of EcCKX see Table 1 in manuscript | |

* Preferentially complete proteins (over 300 aa in length) were chosen for the analysis


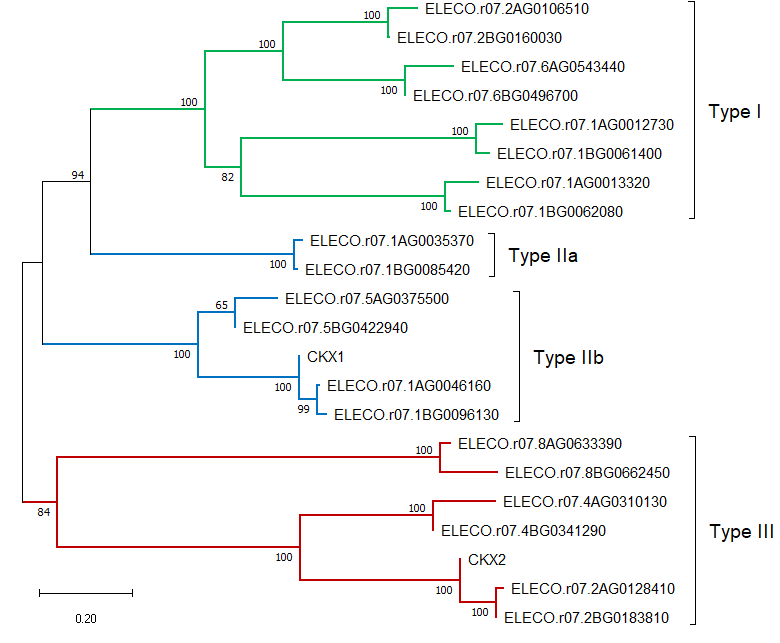


**Supplementary Figure 1.** Phylogenetic tree (NJ) of *E. coracana* *CKX* cds sequences, constructed with 1000 replicates bootstrap support. Initial identification of CKX isotypes and analysis of homologs of previously described *EcCKX1* and *EcCKX2* was conducted basing on the results of this analysis.
